# Development and validation of radiology-clinical statistical and machine learning model for stroke-associated pneumonia after first intracerebral haemorrhage

**DOI:** 10.1186/s12890-024-03160-0

**Published:** 2024-07-24

**Authors:** Wenru Zhang, Ying Zhou, Liuhui Xu, Chaomin Qiu, Zhixian Luo, Zhenghao Jiang, Xinyi Tao, Yingjie Wu, Shishi Yao, Hang Huang, Xinshi Wang, Yunjun Yang, Ru Lin

**Affiliations:** 1 Department of Radiology, The First Affiliated Hospital of Wenzhou Medical University, Wenzhou, Zhejiang China; 2 Wenzhou Medical University, Wenzhou, Zhejiang China; 3 Department of Neurology, The First Affiliated Hospital of Wenzhou Medical University, Wenzhou, Zhejiang China; 4 Key Laboratory of Alzheimer’s Disease of Zhejiang Province, Institute of Aging, Wenzhou Medical University, Wenzhou, Zhejiang China

**Keywords:** Intracerebral haemorrhage, Pneumonia, Cerebral vascular disease, Machine learning, Risk factor

## Abstract

**Background:**

Society is burdened with stroke-associated pneumonia (SAP) after intracerebral haemorrhage (ICH). Cerebral small vessel disease (CSVD) complicates clinical manifestations of stroke. In this study, we redefined the CSVD burden score and incorporated it into a novel radiological-clinical prediction model for SAP.

**Materials and methods:**

A total of 1278 patients admitted to a tertiary hospital between 1 January 2010 and 31 December 2019 were included. The participants were divided into training and testing groups using fivefold cross-validation method. Four models, two traditional statistical models (logistic regression and ISAN) and two machine learning models (random forest and support vector machine), were established and evaluated. The outcomes and baseline characteristics were compared between the SAP and non-SAP groups.

**Results:**

Among the of 1278 patients, 281(22.0%) developed SAP after their first ICH. Multivariate analysis revealed that the logistic regression (LR) model was superior in predicting SAP in both the training and testing groups. Independent predictors of SAP after ICH included total CSVD burden score (OR, 1.29; 95% CI, 1.03–1.54), haematoma extension into ventricle (OR, 2.28; 95% CI, 1.87–3.31), haematoma with multilobar involvement (OR, 2.14; 95% CI, 1.44–3.18), transpharyngeal intubation operation (OR, 3.89; 95% CI, 2.7–5.62), admission NIHSS score ≥ 10 (OR, 2.06; 95% CI, 1.42–3.01), male sex (OR, 1.69; 95% CI, 1.16–2.52), and age ≥ 67 (OR, 2.24; 95% CI, 1.56–3.22). The patients in the SAP group had worse outcomes than those in the non-SAP group.

**Conclusion:**

This study established a clinically combined imaging model for predicting stroke-associated pneumonia and demonstrated superior performance compared with the existing ISAN model. Given the poor outcomes observed in patients with SAP, the use of individualised predictive nomograms is vital in clinical practice.

**Supplementary Information:**

The online version contains supplementary material available at 10.1186/s12890-024-03160-0.

## Background

Intracerebral haemorrhage (ICH) ranks among the top causes of stroke-related morbidity and mortality globally, comprising 15% of all stroke cases [1–3]. Stroke-associated pneumonia (SAP), a common complication, worsens stroke outcomes, extending hospital stays, escalating healthcare costs, and increasing long-term mortality, particularly in affected patients [4–12].

Past research has identified several risk factors linked to SAP onset, including advanced age [8, 9, 13–16], male gender [8, 13, 15, 17], hypertension [18], heart failure [16, 17, 19], severe stroke [8, 13, 16, 20], dysphagia [14–16, 18, 21], elevated neutrophil-to-lymphocyte ratio [22], and high admission blood glucose levels [16, 23]. Several risk scores have been established based on the routinely collected parameters. For instance, the risk between ISAN scoring layered ischemia and hemorrhagic stroke, while A^2^DS^2^ and AIS-APS scores specially used for ischemic stroke depend on the clinical characteristics of easy use [1, 7, 15, 16]. However, these studies lack sufficient radiology and targeted prediction factors.

Cerebral and brain-brain small vascular disease (CSVD) is a chronic deductive cerebral vascular condition, which affects the global function and structure of the brain [24–28]. Dysfunction of endothelial cells, leading to blood–brain barrier dysfunction, disruption of blood flow homeostasis, and abnormal inflammatory responses, have been recognized as both the initial driver of CSVD and factor affecting systematic vascular inflammation, including pulmonary microvascular inflammation [29, 30]. Previous studies have shown that after ICH, there is positive correlation between CSVD burden, increased haematoma, and poor results [31–34]. A survey conducted by YY (2022) shows that combining CSVD's neural imaging features with A^2^DS^2^ scores is a promising method for predicting SAP and bad ending of patients with acute cerebral infarction [35]. However, the association between the CSVD burden and the occurrence of SAP after ICH remains to be explored.

Therefore, in this study, we aimed to (1) evaluate whether CSVD burden independently contributes to SAP development after first ICH, (2) implement an image scoring system to quantify the CSVD burden, and (3) develop and validate a radiology-clinical model for predicting SAP risk.

## Methods

### Patients and follow-up

**Fig. 1:**
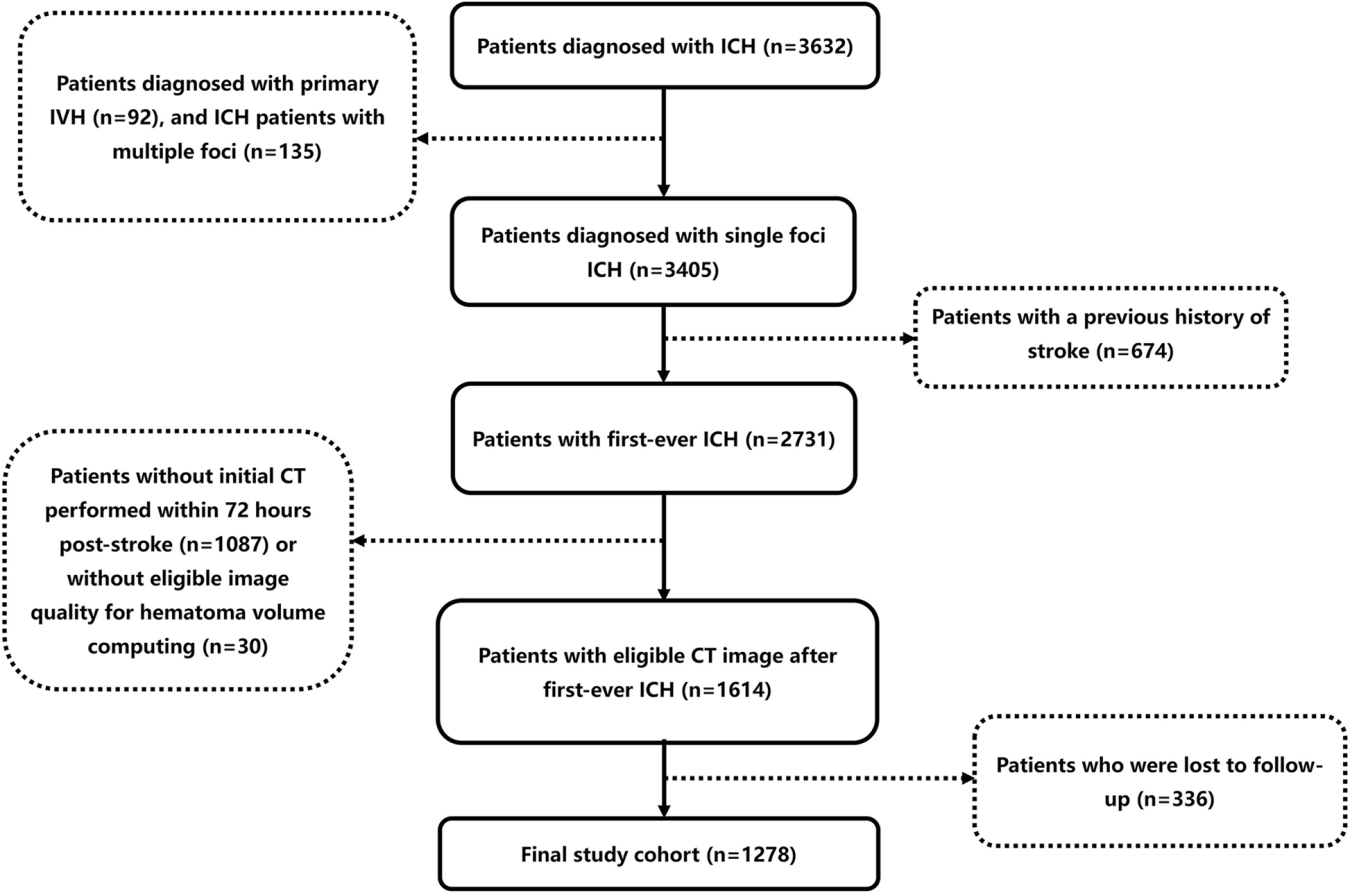
Patient recruitment flowchart. ICH, intracerebral hemorrhage; IVH, intraventricular hemorrhage; CT, computed tomography

All 1278 patients consecutively enrolled in this retrospective study were diagnosed with ICH. The diagnosis was confirmed using computed tomography (CT) at our institution. They were admitted to a tertiary hospital between 1 January 2010 and 31 December 2019. The exclusion criteria included: (1) patients diagnosed with primary intraventricular haemorrhage and/or those with multiple ICH foci resulting in difficulties in calculating haematoma volume; (2) patients with a history of stroke, possibly with complete or partial paralysis; (3) patients who did not undergo CT within 72 h post-stroke or those who did not have CT scans with required image quality for calculating haematoma volume; and (4) patients who were lost to follow-up or declined to participate in the study (see Fig. 1). Participant deaths were recorded using an electronic medical system and were supplemented by telephone interviews.

### Clinicodemographic variables

Data on age, sex, current smoking status, and alcohol consumption were also collected. The coexisting diseases included hypertension, diabetes mellitus, ischaemic heart disease, atrial fibrillation, hyperlipidaemia, and hyperuricaemia. Additional clinical characteristics such as neutrophil-to-lymphocyte ratio, blood pressure, and National Institutes of Health Stroke Scale (NIHSS) score were also recorded upon admission. The body mass index (BMI) was also recorded. Other potential predictors, including post-stroke vomiting, dysphagia (evaluated as dysphagia or requiring dysphagia rehabilitation training), transpharyngeal intubation (orogastric, nasogastric, and endotracheal tubes), and post-stroke pump proton inhibitor (PPI) usage, were confirmed through the electronic medical system.

### Radiological variables

The volume and location of the ICH were obtained and verified using CT within 72-h of stroke onset. Standardised window widths and levels were applied to the CT images to distinguish the haematomas from the brain tissue. The haematoma volume was measured using a manually outlined haematoma profile on each slice of non-enhanced CT (3D Slicer software version 4.10.2). The haematoma contours of each patient were delineated independently by two radiologists blinded to the clinical data (RL and RW). A senior radiologist (YY) was consulted to reach consensus when the contours differed. The location of the ICH was categorised as follows: cortical involvement (defined as any haemorrhage involving the cortex), deep involvement (including the basal ganglia and thalamus), infratentorial involvement (including the brainstem and cerebellum), and multilobar involvement (defined as a haematoma involving two or more lobes). CSVD burden included white matter lesions and cortical/central brain atrophy. The severity of the white matter lesions was assessed using the sum of the anterior/posterior white matter CT scores (0 none, 1–2, mild, 3–4 is severe) [36]. The degree of brain atrophy was measured using the intercaudate distance to inner table width ratio (CC/IT) and temporal horn to choroid fissure distance [37, 38]. The total CSVD burden score was determined by collectively considering the score of CC/IT (≥ cutoff value 0.15, 1 point), the temporal horn to choroid fissure distance (≥ cutoff value 0.46, 1 point), and white matter lesion CT score (mild 1 point, severe 2 points).

### Primary and secondary outcomes

ICH-associated pneumonia (ICH-SAP) was determined according to the SAP consensus (defined as the spectrum of lower respiratory tract infections within the first seven days after stroke onset) [39]. To avoid false positive outcome, SAP in the study was also verified by the following criteria: (1) absence of infection within two weeks before stroke onset; (2) diagnosis of pneumonia based on a combination of clinical presentation (fever, cough, etc.), positive laboratory findings (white blood cell count ≥ 11*10^9/L, neutrophil count ≥ 7.5*10^9/L etc.), and positive chest CT findings; (3) initiation of antibiotic therapy after pneumonia diagnosis. To compare the efficacies of ICH-SAP prediction between our model and the ISAN model [7], the ICH-SAP probability based on the ISAN model was calculated and recorded. Other secondary outcomes included hospitalisation duration, modified Rankin scale (mRS) score at discharge, and all-cause mortality within 30 or 90 days after discharge.

### Statistical analysis

**Fig. 2:**
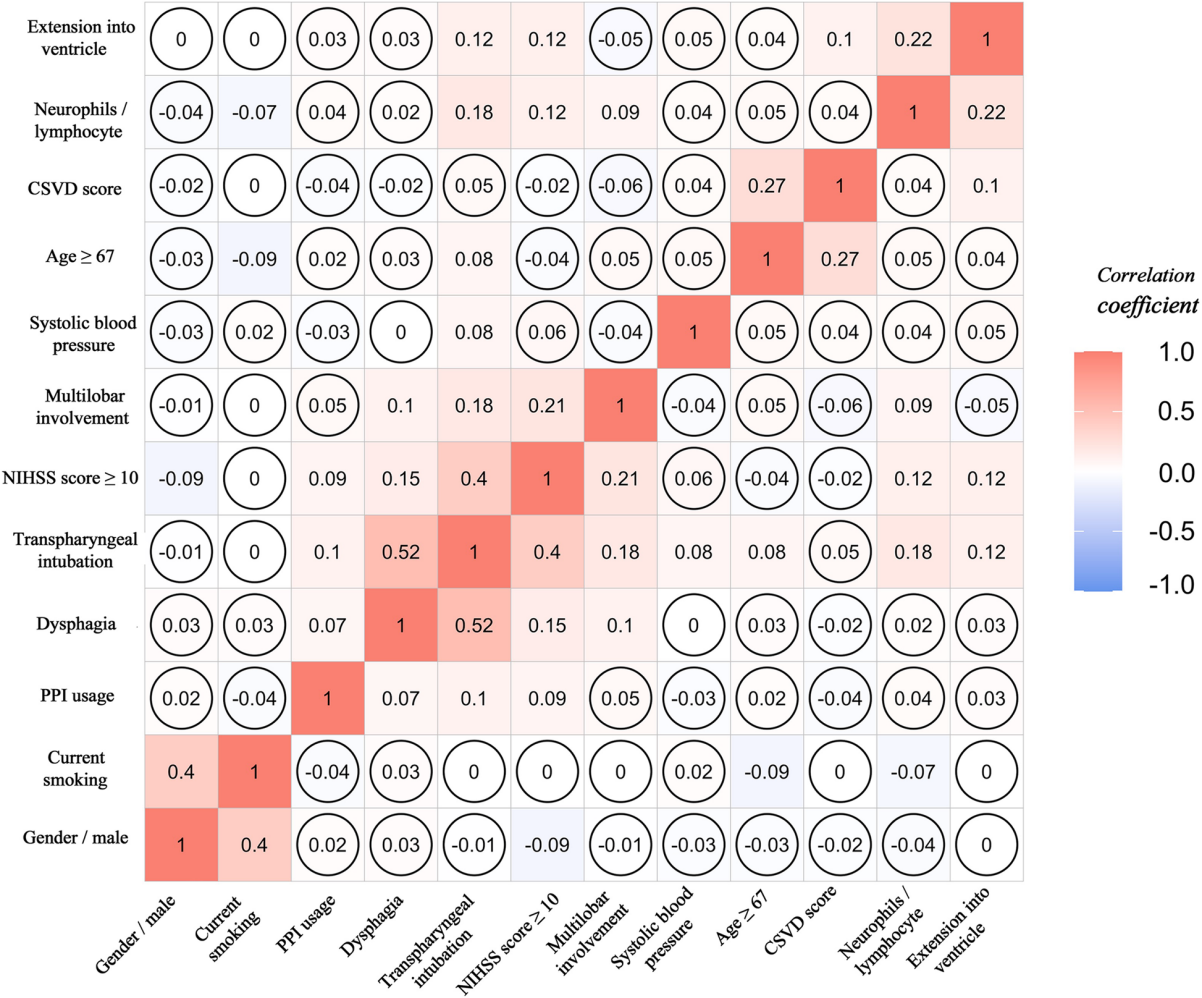
Heatmap for correlation analysis within variables significant in univariable analysis. Different colors filling in the square represented different correlation coefficients. The square with a circle inside it represented a non-significant correlation between two corresponding variables. Numbers in the square represented the correlation coefficient. CSVD, cerebral small vessel disease; NIHSS, National Institutes of Health Stroke Scale; PPI, proton pump inhibitor

In the univariate analysis, independent t-tests (for variables with a normal distribution) or Mann–Whitney U tests (for variables with a non-normal distribution) were used to compare continuous variables, whereas the chi-square test or Fisher’s exact test was used for categorical variables between the SAP and non-SAP groups. The optimal cutoff value was selected by maximising the Youden index. Correlation analysis was performed among variables with a two-sided *p*-value < 0.1 and depicted using a correlation analysis heatmap (see Fig. 2).

### Model development

The training and validation groups were created using a fivefold cross-validation method with 400 repetitions, ensuring that the sampling was proportional to the original dataset. Logistic regression (LR) was employed alongside two machine learning (ML) methods, namely, support vector machine (SVM) and random forest (RF), to establish the SAP prediction model. A grid search method was used to identify the best gamma and cost coefficients in the SVM model ( Figure S1). The ‘best_mtry’, ‘best_ntree’, ‘best_maxnodes’ and ‘best_nodesize’ were determined to establish a reasonable RF model with hyperparameter optimisation (see Figure S2). Moreover, in developing the LR model, multivariate analysis was conducted using a forward stepwise regression approach with the maximum likelihood ratio method to assess the independent predictors of SAP.

### Model evaluation

The area under the curve (AUC) and its corresponding 95% confidence interval (95% CI) were calculated and are shown in Table 2. The probability of SAP risk obtained from the ISAN scoring system was used to calculate the corresponding AUC value. Furthermore, based on the results of the LR model, a forest plot was generated to illustrate the odds ratios (OR) and their 95% confidence intervals (CI) for independent predictors of SAP, and a nomogram with a gradient colour style was created to calculate individualised ICH-SAP risk. Additionally, a calibration curve with 80–95% CI was generated for both the training and testing groups.

### Outcome analysis

Differences in hospitalisation duration, mRS ≥ 3 at discharge, and all-cause mortality of 30 days or 90 days after discharge between the SAP and non-SAP groups were compared. All statistical analyses were conducted using the R software (Version 4.1.2). The packages utilised in the analysis are described in the Supplementary Methods.

## Result

### Baseline patient characteristics

Among the 1278 patients finally enrolled in the study, the SAP occurrence rate after the first ICH was 22.0% (*n* = 281/1278). Thirty one point seven percent (*n* = 405/1278) of patients aged ≥ 67 years(The cutoff value calculated based on the Youden index.). Seventy percent of patients the total cohort were male. The other characteristics of the cohort are shown in Table S1.

### Results of univariable analysis

Age ≥ 67 years, dysphagia, transpharyngeal intubation, neutrophils-to-lymphocyte ratio, admission NIHSS score ≥ 10, multilobar involvement, haemorrhage extension into the ventricle, and high CSVD burden score were statistically different between patients with and without SAP (*p* < 0.001). Patients with SAP were more likely to have a higher systolic blood pressure on admission (164.83 vs. 160.15, *p* = 0.044). Additionally, the proportions of male patients and current smokers were higher in the SAP group (74.4% vs. 68.7%, *p* = 0.067; and 33.8% vs. 28.1%, *p* = 0.063, respectively), although the difference was not statistically significant (Table 1). All variables mentioned above (*p* < 0.1) in the univariable analysis were further included in the correlation and model establishment analyses.

**Table 1:** Predictors of SAP: univariable analysis

Variables	SAP(*n* = 281)	Non-SAP(*n* = 997)	*P*-value
**Demographics**			
Age ≥ 67	45.9% (*n* = 129/281)	27.7% (276/997)	0.000
Gender (male/total)	74.4% (*n* = 209/281)	68.7% (*n* = 685/997)	0.067
**Lifestyle-related variables**			
Current drinking	35.9% *(n = 101/281)*	34.9% (*n* = 348/997)	0.747
Current smoking	33.8% (*n* = 95/281)	28.1% (*n* = 280/997)	0.063
**Clinical variables**			
post-stroke vomiting	27.9% (*n* = 78/280)	24.6% (*n* = 245/996)	0.268
Dysphagia	17.5% (*n* = 48/275)	7.4% (*n* = 73/980)	0.000
Transpharyngeal intubation	59% (*n* = 164/278)	19.7% (*n* = 194/984)	0.000
Post-stroke PPI usage	46.7% (*n* = 128/281)	37.1% (*n* = 364/997)	0.004
**Coexisting disease**			
Hypertension	90.7% (*n* = 255/281)	90.3% (*n* = 900/997)	0.811
Diabetes mellitus	18.9% (*n* = 53/281)	19.8% (*n* = 197/997)	0.737
Ischemic heart disease	5.3% (*n* = 15/281)	5.6% (*n* = 56/997)	0.857
Atrial fibrillation	3.2% (*n* = 9/281)	1.8% (*n* = 18/997)	0.150
Hyperlipidemia	22.8% (*n* = 64/281)	26.7% (*n* = 266/997)	0.187
Hyperuricemia	5.0% (*n* = 14/281)	4.5% (*n* = 45/997)	0.741
**Laboratory index**			
BMI index, n (%)			0.297
< 18.5	9.9% (*n* = 8/81)	5.5% (*n* = 23/416)	
18.5–24	40.7% (*n* = 33/81)	45.7% (*n* = 190/416)	
≥ 24	49.4% (*n* = 40/81)	48.8% (*n* = 203/416)	
Neutrophils/lymphocyte, median (IQR)	6.09 (10.14, 3.9)	4.25 (2.67, 7.57)	0.000
Admission systolic blood pressure, mean ± SD	164.83 ± 25.85	160.15 ± 24.12	0.044
Admission diastolic blood pressure, median (IQR)	91 (80, 100)	90 (80, 100)	0.812
Discharge mRS ≥ 3	81.5% (*n* = 229/281)	51.2% (*n* = 510/997)	0.000
Hospitalization duration (day), median (IQR)	17 (13, 22)	14 (11, 18)	0.000
Death within 30-day discharge	0.7%(*n* = 2/281)	0.3%(*n* = 3/997)	0.665
Death within 90-day discharge	2.1% (*n* = 6/281)	0.8% (*n* = 8/997)	0.116
NIHSS score ≥ 10	59.1% (*n* = 166/281)	29.4% (*n* = 293/997)	0.000
**Radiological variables**			
Haematoma volume (ml), median (IQR)	7.83(2.94,15.27)	7.62(3.22,14.22)	0.789
Cortical involvement	35.2% (*n* = 99/281)	32.8% (*n* = 327/997)	0.445
Deep involvement	78.3% (*n* = 220/281)	75.4% (*n* = 752/997)	0.320
Infratentorial involvement	8.9% (*n* = 25/281)	12.0% (*n* = 120/997)	0.166
Multilobar involvement	34.2% (*n* = 96/281)	15.5% (*n* = 155/997)	0.000
Extension into ventricle	35.6% (*n* = 100/281)	19.4% (*n* = 193/997)	0.000
Total CSVD burden score	1 (1, 2)	1 (1, 2)	0.000
Follow-up duration (day), median (IQR)	1624 (1065, 2544)	1945 (1274, 2715)	0.000

*SD* Standard Deviation, *IQR* Interquartile Range, *PPI* proton pump inhibitor, *BMI* Body Mass Index, *mRS* modified Rankin scale, *NIHSS* National Institutes of Health Stroke Scale, *CSVD*, cerebral small vessel disease; Continuous variables were expressed as mean ± standard deviation or median (IQR). Categorical variables were expressed as counts and percentages

### Development, validation, and evaluation of different model

**Fig. 3:**
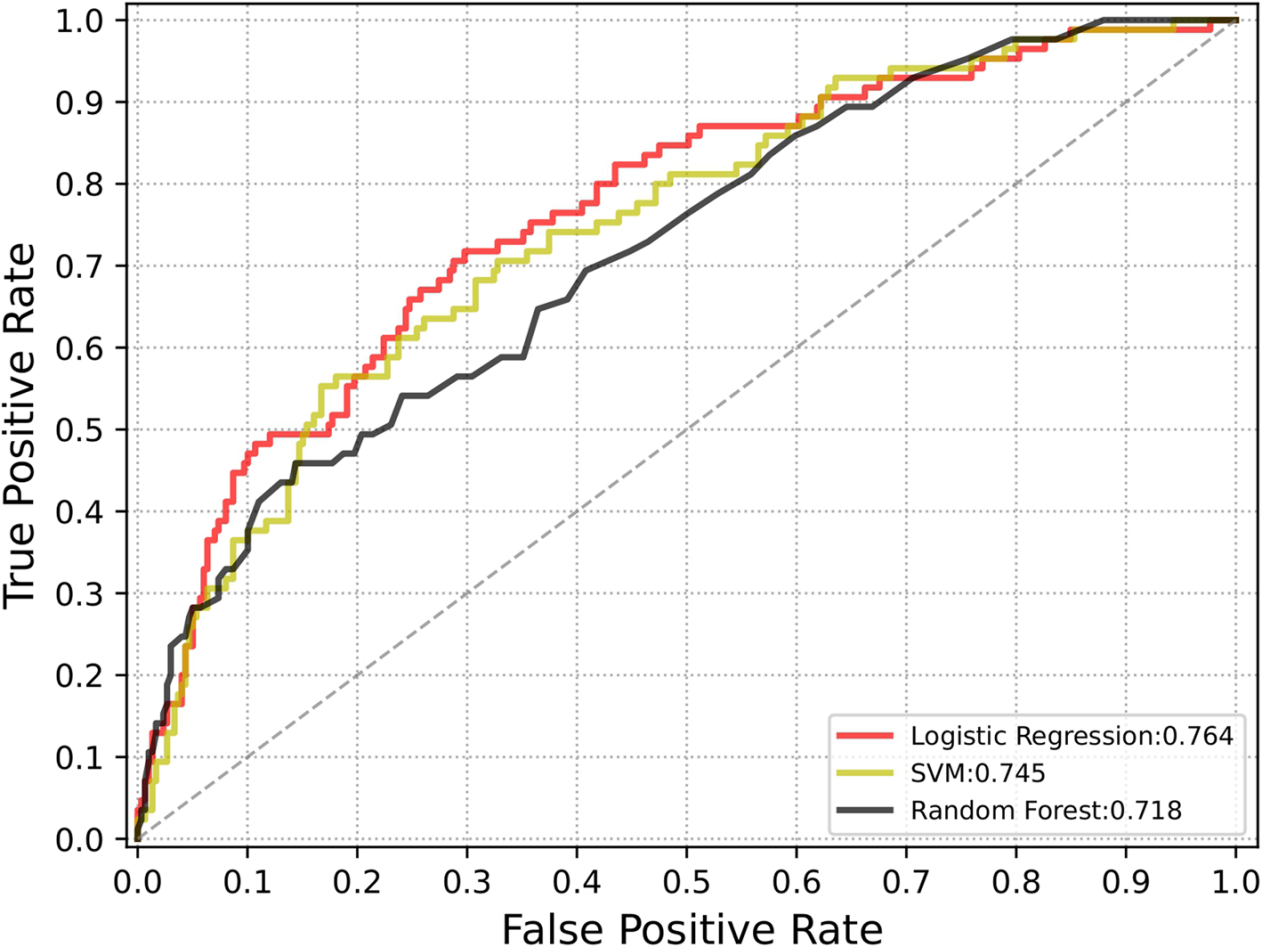
ROC curve of different models in the cohort group. AUC, area under the curve

**Fig. 4:**
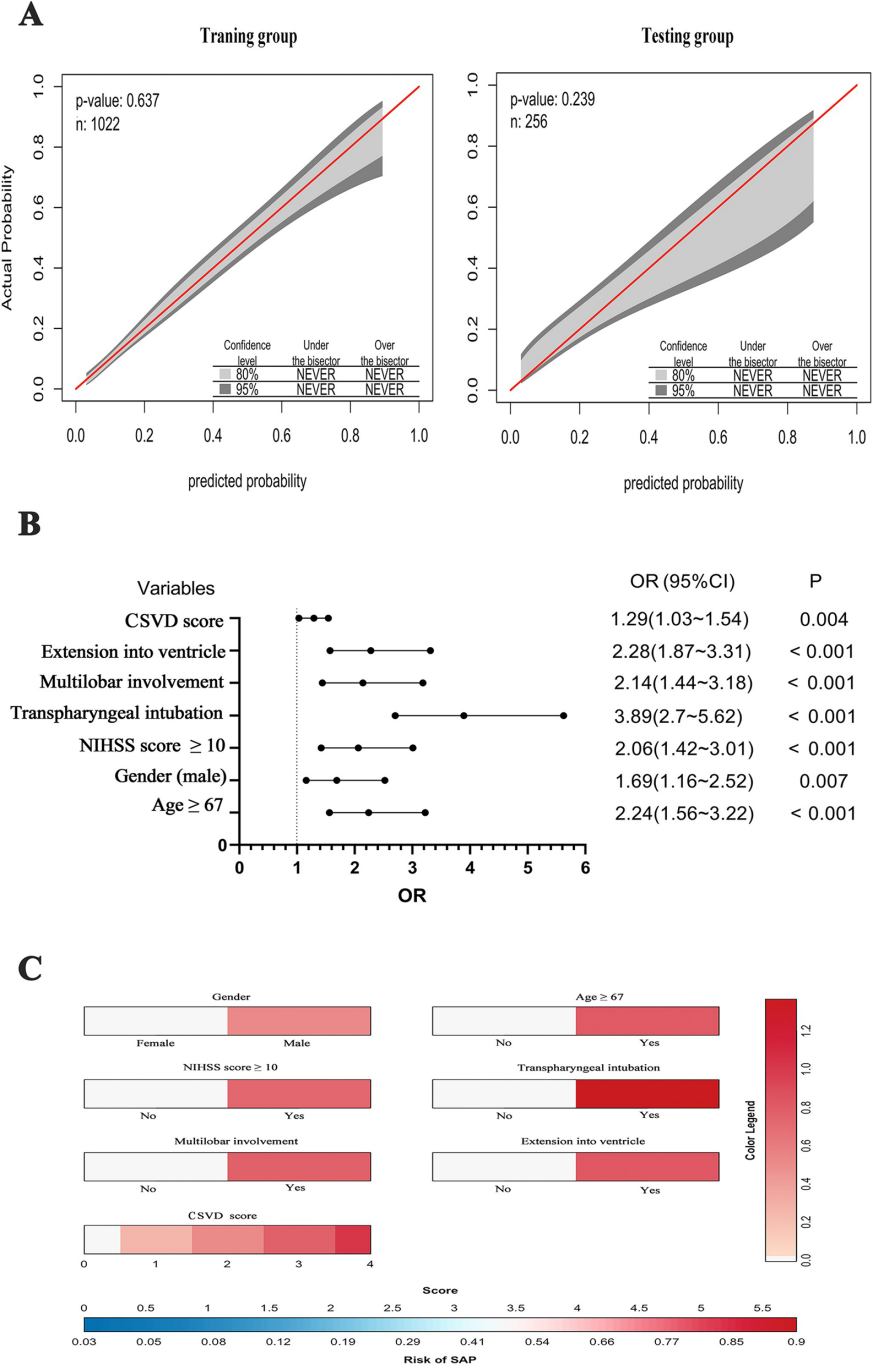
Information of multivariable logistic regression model. **A** displayed the calibration curve belt of the logistic regression model in the training and testing groups, respectively. The gray belt region represented a well-calibrated situation of both training and testing groups within an 80% to 95% confidence interval range. **B** was a forest plot displaying the results of the multivariable analysis. **C** was a nomogram with gradient colors representing different total scores and SAP risk. CSVD, cerebral small vessel disease; NIHSS, National Institutes of Health Stroke Scale; OR, odds ratio; 95% CI, 95% confidence interval; SAP, stroke-associated pneumonia

The variables used to build the SVM and RF models were not highly correlated (Fig. 2). The classified SVM model was constructed using a radial kernel with the best gamma (0.1) and cost (1) parameters. The AUC for the training and testing groups in the SVM model were 0.812 and 0.674, respectively (Table 2). The RF model was established with parameters ‘mtry’, ‘ntree’’, maxnodes’, and ‘nodesize’ equal to 10, 580, 7, and 3, respectively (see Figure S2). The AUC of the values for the training and testing groups were 0.684 and 0.652, respectively (Table 2). The final LR model indicated that total CSVD burden score (OR, 1.29; 95% CI, 1.03–1.54; *p* = 0.004), haematoma extension into ventricle (OR, 2.28; 95% CI, 1.87–3.31; *p* < 0.001), haematoma with multilobar involvement (OR, 2.14; 95% CI, 1.44–3.18; *p* < 0.001), transpharyngeal intubation operation (OR, 3.89; 95% CI, 2.7–5.62; *p* < 0.001), admission NIHSS score ≥ 10 (OR, 2.06; 95% CI, 1.42–3.01; *p* < 0.001), male sex (OR, 1.69; 95% CI, 1.16–2.52; *p* = 0.007), and age ≥ 67 (OR, 2.24; 95% CI, 1.56–3.22; *p* < 0.001) were independent predictors of SAP after ICH (see Fig. 4B). The AUC for the training and testing groups in the LR model were 0.796 and 0.746, respectively (Table 2). Figure 3 shows a comparison of the AUC values for the cohort groups of the three models. Figure 4A indicates that the LR model did not overestimate or underestimate the SAP risk at 80% CI and 95% CI in either the training or testing groups. A practical nomogram for predicting the probability of developing SAP was created (Fig. 4C). The AUC of the ISAN model based on the ISAN SAP risk probability was 0.688. The sensitivity, specificity, positive predictive value (PPV), negative predictive value (NPV), F1 score, and accuracy of each model are presented in Table 2.

**Table 2:** Multiple model evaluation indexes for different model

Model evaluation index	Support vector machine		Random forest		Logistic regression		ISAN
	Training group	Testing group	Training group	Testing group	Training group	Testing group	Total group
AUC	0.812	0.674	0.684	0.652	0.796	0.746	0.688
95% CI	0.783–0.842	0.602–0.747	0.650–0.718	0.580–0.724	0.762–0.830	0.677–0.815	——
Sensitivity	0.793	0.395	0.471	0.463	0.387	0.395	0.149
Specificity	0.832	0.874	0.897	0.841	0.940	0.974	0.904
Positive predictive value	0.573	0.593	0.566	0.439	0.644	0.593	0.304
Negative predictive value	0.934	0.756	0.856	0.854	0.844	0.756	0.790
F1 score	0.665	0.474	0.514	0.450	0.483	0.474	0.200
Accuracy	0.823	0.722	0.803	0.761	0.818	0.722	0.527

### Results of short and long-term outcomes analysis

**Fig. 5:**
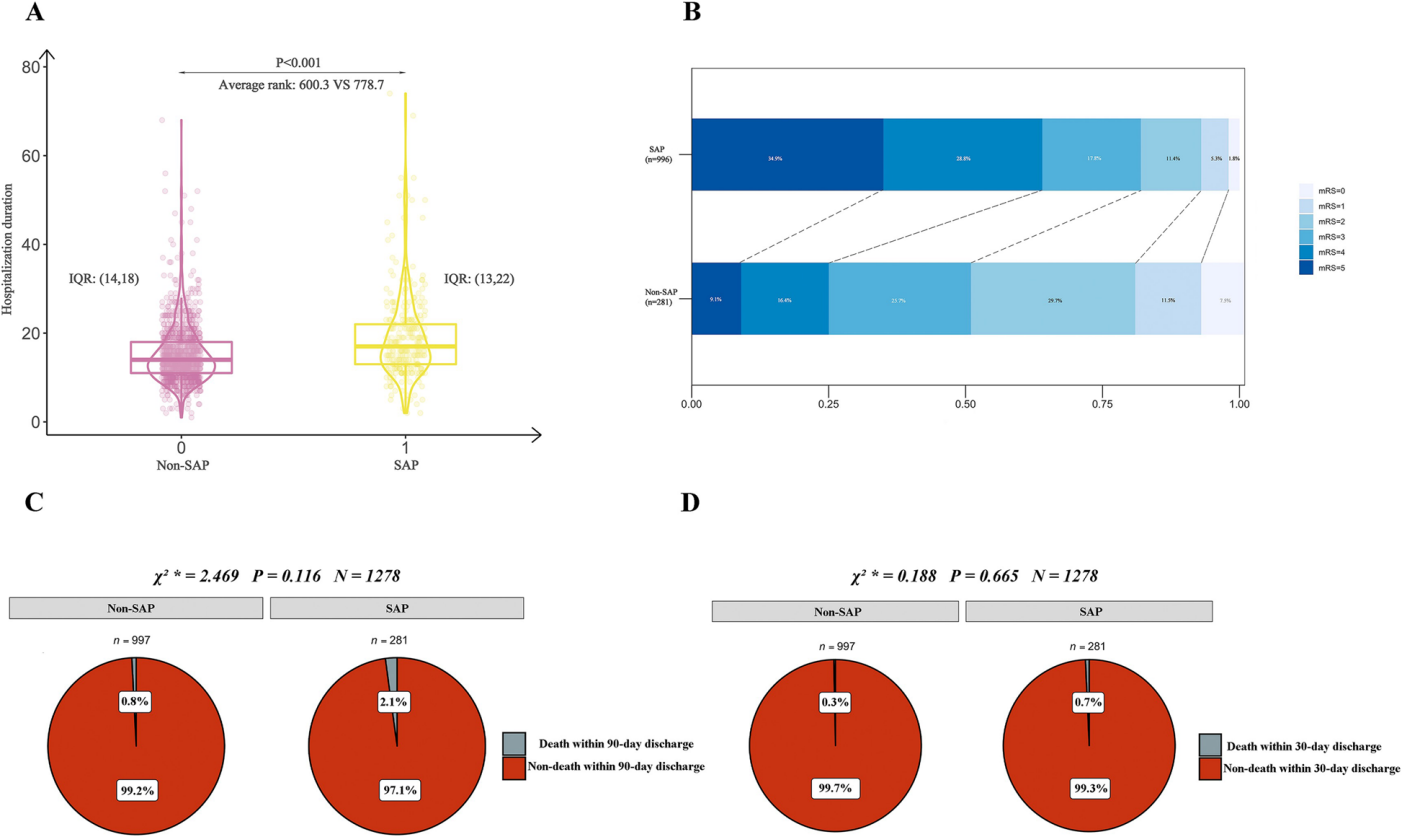
Short or long-term prognosis difference between SAP and non-SAP patients. **A** showed the difference in hospitalization duration between patients with and without SAP using Mann–Whitney u test. **B** displayed the proportion of the SAP or non-SAP patient’s mRS at discharge. **C** and (**D**) were comparison results of death within 90-day or death within 30-day discharge difference between SAP and non-SAP group. SAP, stroke-associated pneumonia; IQR, interquartile range; mRS, modified Rankin scale

Patients with SAP had a significantly longer duration of hospitalisation than those without SAP (*p* < 0.001). Moreover, patients with SAP exhibited a higher frequency of poor outcomes, defined as mRS ≥ 3 at discharge, compared to that of patients without SAP (81.5% vs. 51.2%, *p* < 0.001, Table 1). Additionally, the 30-day or 90-day discharge mortality rates were higher in the SAP group than in the non-SAP group (0.7% vs. 0.3%, *p* = 0.665, and 2.1% vs. 0.8%, *p* = 0.116, respectively). The detailed distribution of functional outcomes at the time of discharge in patients with and without SAP is presented in Fig. 5.

## Discussion

Our study indicated that the multivariable logistic regression model achieved superior SAP prediction ability compared to both the ML models and the existing ISAN model. The following risk factors: age ≥ 67 years, male sex, transpharyngeal intubation, NHSS score ≥ 10 on admission, haematoma involving multiple lobes or extending into ventricle, and high total CSVD burden score were identified as independent predictors of SAP after first ICH.

Advanced age, male gender, significant neurofunctional deficits, and extension of the haematoma into the ventricle were predictive factors for SAP occurrence, aligning with previously reported results [8, 14–17]. The presence of multilobar involvement in the haematoma, indicating a larger haematoma volume, strongly correlated with SAP development in our study. This finding supports previous research, which showed that patients with a 1 ml increase in hematoma volume were 1.02 times more likely to develop SAP compared to those without it [1]. Our study demonstrated that transpharyngeal intubation, an easily judged and accessible indicator in clinical practice, independently increased the risk of SAP, whereas dysphagia did not reach statistical significance in the final model after controlling for confounding factors. This may be attributed to the fact that manipulating the transoropharyngeal route increases the risk of accidental aspiration.

CSVD has been extensively studied, whereas the association between CSVD and SAP has rarely been discussed [24, 25, 27, 28, 31, 32]. A previous study hypothesised that older patients with a history of severe cerebral atrophy were likely to develop lower respiratory tract infections [40]. Nam et al. found that patients with severe leukoaraiosis were likely to develop SAP after acute ischaemic stroke, which may be accounted for by studying the correlation between the degree of leukoaraiosis and the level of impaired brain structure and function (such as reduction of the cough reflex) [41]. Another study indicated that brain atrophy may contribute to the occurrence of SAP through indirect influence on swallowing reflex and that dilated perivascular spaces interpreted as blood–brain barrier dysfunction are associated with poor outcomes [35]. The degree of CSVD burden, which represents the level of endothelial cell dysfunction in microvascular inflammation, results in a highly inflammatory microenvironment in the pulmonary glands [29, 42, 43]. In this study, CSVD was found to be independently associated with the development of SAP after ICH, after controlling for haematoma volume confounders. This relationship may be explained by several hypotheses. First, in line with existing assumptions, CSVD is always accompanied by impairment of neurofunction, indirectly affecting SAP occurrence to some extent. Second, CSVD, deemed as endothelial cell activation and dysfunction, accelerates the formation of the pulmonary inflammatory microenvironment, contributing to the development of SAP. Therefore, the possible cumulative effect of the CSVD burden on patients likely predicts ICH-SAP risk and helps in the clinical rationalisation of medical resources, designation of care plans, and implementation of targeted preventive strategies.

The ISAN model has demonstrated high clinical applicability in patients with stroke, exhibiting the ability to accurately distinguish between SAP and non-SAP patients [7]. However, this study was primarily conducted in the ischaemic stroke group, with only 8% of the patients having ICH. In addition, this model did not include radiological elements, and the AUC value of the model in our study was only 0.688. The logistic regression model, incorporating both clinical and radiological risk factors; showed good differentiation ability for SAP and was well-calibrated in both our study’s training and testing groups. The high negative predictive value compared with the positive predictive value in our study, coupled with a well-calibrated belt indicating no over-or underestimation of SAP, suggesting that future prognostic models might benefit from attempts to achieve a more balanced distribution of differentiation utility between higher and lower values. Although our study did not include mRS scores at 3-month discharge, we observed that patients who experienced ICH-SAP tended to have longer hospitalisation durations and higher all-cause mortality during the longitudinal follow-up period.

Our study has several limitations. First, given the retrospective nature of the study, SAP may have been underestimated, despite the strict diagnostic criteria used. Additionally, patients with SAP exhibited a tendency towards poor outcomes in this study, although longitudinal mRS score follow-up was not conducted. Therefore, large-scale studies with standardised long-term observations are required. Second, only patients with first-ever ICH were included in this study, potentially leading to a selection bias, as pre-stroke dependence has been shown to be a risk factor for SAP. Furthermore, the radiology-clinical model was not validated using an external dataset. Third, although the underlying mechanism by which CSVD influences ICH-SAP remains unclear, the current study demonstrates that CSVD is a predictive factor for ICH-SAP, providing guidance for future research.

In conclusion, we developed a novel radiological-clinical model to predict SAP after first ICH. Future studies are required to further explore and confirm the relationship between CSVD and SAP.

## Conclusion

This study indicated that CSVD burden increased the risk of SAP after first ICH, independent of ICH volume. The novel radiology-clinical SAP model, incorporating the CSVD burden, was optimally established by logistic regression, surpassing two other machine learning models and the ISAN model in terms of performance. Patients developing SAP tended to have a poor prognosis in short- and long-term follow-ups. A nomogram with a gradient colour style was created based on a well-calibrated model to aid in the early identification of patients at a high risk of ICH-SAP in clinical practice. This tool assists in the selection of appropriate treatment and care strategies, thereby enhancing outcomes and potentially preventing SAP-related complications.

### Supplementary Information

Supplementary Material 1.

## Data Availability

The data supporting this study’s findings are available at reasonable request from the corresponding author.

## Abbreviations

ICH: Intracerebral haemorrhage
SAP: Stroke-associated pneumonia
CSVD: Cerebral small vessel disease
CT: Computed tomography
IVH: Intraventricular haemorrhage
NIHSS: National Institutes of Health Stroke Scale
BMI: Body mass index
PPI: Proton pump inhibitor
ICH-SAP: ICH associated-pneumonia
mRS: Modified Rankin scale
SVM: Support vector machine
RF: Random forest
LR: Logistic regression
AUC: Area under the curve
OR: Odds ratio
95% CI: 95% Confidence interval
PPV: Positive predictive value
NPV: Negative predictive value
