# Supplementary material for: Development and validation of radiology-clinical statistical and machine learning model for stroke-associated pneumonia after first intracerebral haemorrhage
Source: BMC Pulm Med. 2024 Jul 24;24:357. doi: 10.1186/s12890-024-03160-0 (PMC11267827; doi:10.1186/s12890-024-03160-0)
Supplement: Supplementary file 1 — Supplementary Material 1. [file 12890_2024_3160_MOESM1_ESM.pdf]

Development and validation of radiology-clinical statistical and machine learning model for stroke-associated pneumonia after first-ever intracerebral hemorrhage

Supplemental Materials

Statistical analysis

The package ‘foreign’ was used to read data. The package ‘ggcorplot’ was used to compute correlation coefficient and generate a heatmap. The package ‘e1071’ was used to search for the best coefficient value of gamma as well as gamma, and further build a support vector machine. The package ‘randomForest’ was used to establish a randomforest model. The package ‘stats’ was used to build glm model using binominal logit methods. The package ‘caret’ was used to create multi-folds of training as well as testing group, and further computed confusion matrix of three machine learning model, including sensitivity, specificity, Positive predictive value, Negative predictive value, precision, recall, F1 score and accuracy. The package ‘pROC’ was used to compute area under curve value and 95% confidence interval, which then displayed in the receiver and operator curve. The package ‘Giviti’ was used to make the calibration belt. The package ‘regplot’ was used to generate a nomogram with gradient color style. The package ‘ggstatsplot’ and ‘ggplot2’ were used to generate a pie chart using Chi-square analysis methods as well as combination of boxplot, jitter scatter plot and violin-style figure. The package ‘reshape2’, ‘ggplot2’, and ‘ggthemes’ was used to generate bar chart displaying distribution difference of mRS at discharge between SAP and non-SAP group.

Supplementary figures

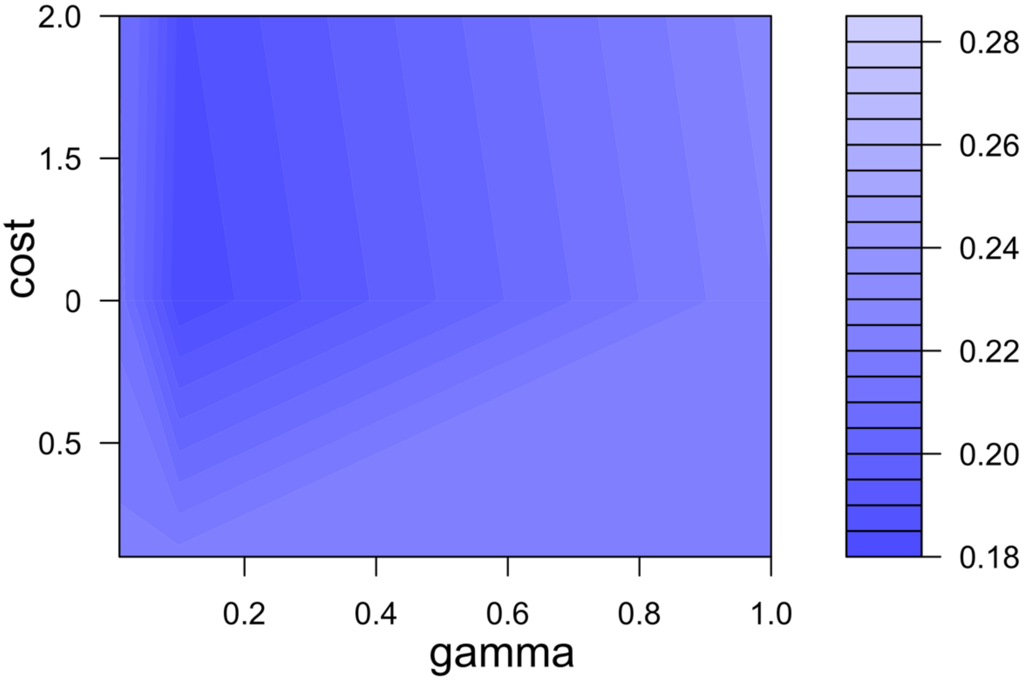

Figure S1. Contour map for grid searching of best gamma and best cost coefficient in the support vector machine hyperparameter optimization process. The darker the color, the better the model optimization.

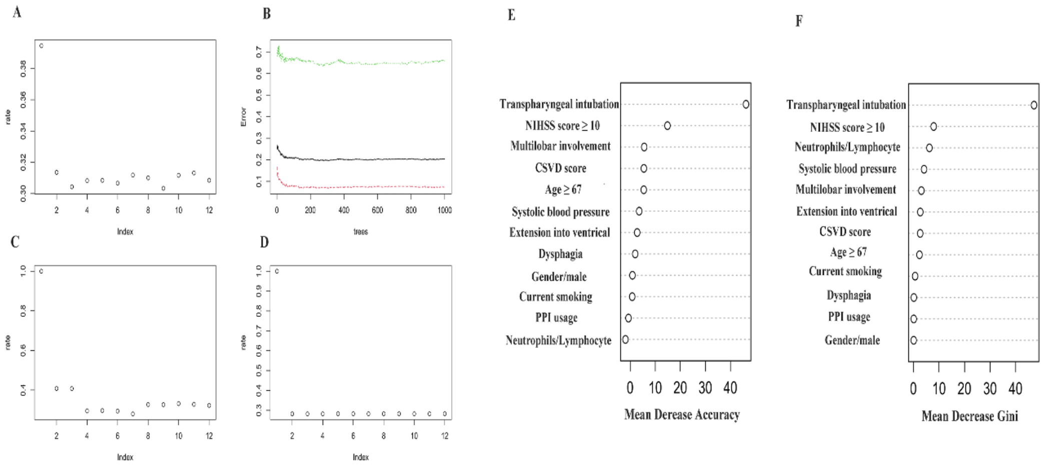

Figure S2. Parameter optimization in the establishment process of random forest model.

Supplementary table

Table S1:Baseline Characteristics on Admission

Variables	Overall cohort(n=1278)
**Demographics**	
Age ≥ 67	31.7% (n=405/1278)
Gender (male/total)	70% (n=894/1278)
**Lifestyle-related variables**	
Current drinking	35.1% (n=449/1278)
Current smoking	29.3% (n=375/1278)
**Clinical variables**	
Post-stroke vomiting	25.3% (n=323/1276)
Dysphagia	9.5% (n=121/1255)
Transpharyngeal intubation	28% (n=358/1262)
Post-stroke PPI usage	38.5% (n=492/1256)
**Coexisting disease**	
Hypertension	90.4% (n=1155/1278)
Diabetes mellitus	19.6% (n=250/1278)
Ischemic heart disease	5.6% (n=71/1278)
Atrial fibrillation	2.1% (n=27/1278)
Hyperlipidemia	25.8% (n=330/1278)
Hyperuricemia	4.6% (n=59/1278)
**Laboratory index**	
BMI index	
<18.5	2.4% (n=31/497)
18.5-24	17.4% (n=223/497)
≥ 24	19% (n=243/497)
Neutrophil / Lymphocyte, median (IQR)	4.7 (2.8, 8.1)
Admission systolic blood pressure, mean ± SD	160.2 ± 23.9
Admission diastolic blood pressure, median (IQR)	90 (80, 100)
Stroke associated Pneumonia (SAP)	22.0% (n=281/1278)
Discharge mRS ≥ 3	56.4% (n=739/1277)
Hospitalization duration (day), median (IQR)	14 (11, 19)
Death within 30-day discharge	0.4%(n=5/1278)
Death within 90-day discharge	1.1% (n=14/1278)
Admission NIHSS score ≥ 10	35.0% (n=459/1278)
**Radiological variables**	
Haematoma volume, median (IQR)	7.59 (3.08,14.43)
Cortical involvement	33.3% (n=426/1278)
Deep involvement	76.1% (n=972/1278)
Infratentorial involvement	11.3% (n=145/1278)
Multilobar involvement	19.6% (n=251/1278)
Extension into ventricle	22.9% (n=293/1278)
CSVD score	1 (1, 2)
Follow-up duration (day), median (IQR)	1882.5 (1209.5, 2683.3)

SD, Standard Deviation; IQR, Interquartile Range; PPI, proton pump inhibitor; BMI, Body Mass Index; mRS, modified Rankin scale; NIHSS, National Institutes of Health Stroke Scale; CSVD, cerebral small vessel disease; Continuous variables were expressed as mean ± standard deviation or median (IQR). Categorical variables were expressed as counts and percentage.
